# Case report: Treatment-resistant depression with acute psychosis in an adolescent girl with Cushing's syndrome

**DOI:** 10.3389/fpsyt.2023.1170890

**Published:** 2023-06-02

**Authors:** Xiaowen Yin, Song Chen, Xuan Ju, Xiwen Hu

**Affiliations:** Hangzhou Seventh People's Hospital, Affiliated Mental Health Center of Zhejiang University School of Medicine, Hangzhou, Zhejiang, China

**Keywords:** Cushing's syndrome (CS), treatment-resistant depression, acute psychosis, adrenal adenoma (AA), adolescent girl

## Abstract

Cushing's syndrome (CS) is a rare disease with multiple somatic signs and a high prevalence of co-occurring depression. However, the characteristics of depression secondary to CS and the differences from major depression have not been described in detail. In this case, we report a 17-year-old girl with treatment-resistant depression with a series of atypical features and acute psychotic episodes, which is a rare condition secondary to CS. This case showed a more detailed profile of depression secondary to CS and highlighted the differences with major depression in clinical features, and it will improve insight into the differential diagnosis especially when the symptoms are not typical.

## Introduction

Depression is a chronic medical problem with typical features, including sadness, decreased interest and cognitive impairments. In clinical practice, depression can occur in other medical conditions, especially endocrinopathies, making it a more complex problem and exhibiting a challenge in diagnosis, especially in first-contact patients or when the clinical presentations are atypical. It is generally accepted that patients who failed to respond to two or more adequate trials of first-line antidepressants for treatment of major depressive episode are considered to have treatment-resistant depression (TRD) (1). For patients with TRD, a throughout evaluation should be performed to investigate the underlying organic causes.

Cushing's syndrome is a rare but serious endocrine disease due to chronic exposure to excess circulating glucocorticoids with multisystem effects (2). The etiology of CS can be divided into adrenocorticotropic hormone (ACTH)-dependent and ACTH-independent. It is characterized by a series of clinical features suggesting hypercortisolism, for example, metabolic abnormalities, hypertension and bone damages (3). A variety of neuropsychiatric symptoms, such as mood disturbance, cognitive impairment and psychosis, also occur in more than 70% CS patients (4). CS is life-threating if not timely diagnosed and treated, however, correct diagnosis can be delayed due to the wide range of phenotypes, especially when they are not classical (5).

Previous studies suggested that major depression was the most common co-morbid complication in CS patients, with a prevalence of 50–81% (6). Haskett's study confirmed that 80% of subjects meet the criteria for major depression with melancholic features (7). As reported in most recent investigations, depression in CS was not qualitatively different from non-endocrine major depression and the similarity was even striking (3, 8). However, some studies showed different conclusions and suggested a high prevalence of atypical depressive features other than melancholic features in CS (9). TRD and anxious depression has also been reported in CS patients (10, 11). All of the above conclusions suggest the complexity of depression with CS, and no distinct features have been found pertaining to hypercortisolism (12, 13). Although the intensity of depression secondary to CS is severe, suicidal depression is still an unusual condition (14).

Psychosis is a rare manifestation of CS, and the literature is limited. Only a few cases have been reported so far, especially combined with depression episode. In this case report, we presented a girl with CS, who experienced suicidal depression with a series of atypical features and acute psychotic symptoms, which was rarely reported in previous studies.

## Case description

A 17-year-old girl with major depression for 3 years was involuntarily admitted for severe depressed mood with suicide attempts (neck cutting; tranquilizer overdose) and paranoid state in the last 2 weeks without any precipitating factors.

She experienced depressed and irritable mood in the last 3 years, and her condition had not improved although several adequate trials of antidepressants were used with satisfactory compliance (sertraline 200 mg/d; escitalopram oxalate 20 mg/d). Over the 2 weeks prior to admission, her depression continued to worsen with increasing irritability, she committed several suicide attempts, and once stated that she was unsafe at home. On admission, her heart rate was 116 bpm with blood pressure 139/81 mmHg and normal temperature; physical examination showed a cushingoid and virilising appearance (central obesity, swollen and hirsute face with acne, purple striae on the abdomen and bruises on the arms). No other abnormal signs were noted. She seemed drowsy but arousable, and she walked slowly, with bent shoulders and an inclined head. Mental state examination was hard to continue because she was passive and reluctant to answer our questions. Venlafaxine 150 mg/d has been used for more than 3 months with poor effects at that time.

Besides, weight gain (25 kg), irregular menstrual cycles and numbness of the hands and feet in the last half year were reported by her parents. Otherwise, No episodes of elevated mood and hyperactivity were found during the history taking. She does not have remarkable family history of serious physical or psychiatric illness; she was healthy, had an extroverted personality and had never used substances. Her premorbid social function and academic performance were good.

Several clinical characteristics found during the following mental state examinations were listed as follows:

- Prominent cognitive impairment without clouding of consciousness: Forgetfulness was frequently noted; she easily forgot important personal information such as her school and grade; she could not recall the suicide attempt committed recently and perfunctorily ascribed it to a casual event; and it was hard for her to recall her medical history (as it is for other depressive patients). The serial seven subtraction task could not be finished, and the interpretation of the proverb was superficial. Difficulty was found in attention maintenance; an effective conversation was hard to perform because she was mind-wandering (we needed to call her name to get her immediate attention) and often interrupted our conversations by introducing irrelevant topics or leaving without apparent reasons.
- Decreased language function that did not match her educational background: The patient could not find the proper words to articulate her feelings; instead, many simple, obscure and contradictory words were used, which made her response seem perfunctory. For example, she responded with “I do not know,” “I forgot,” or kept silent in response to our questions, which made the conversations hard to perform.
- Psychotic outbursts: Once she left the psychological therapy group, ranted about being persecuted and shook in fearfulness, stated “call the police” repeatedly, negative of explanations and comforts from others, but she cannot give any explanation about her behavior when calmed down. Sometimes she worried about being killed by the doctors but the worries were transient and fleeting.
- Depressed mood and negative thoughts (self-blame, worthlessness, and hopelessness) that were not persistent and profound: During most of her hospitalization, the patient seemed confused and apathetic, with intermittent anxiety, but she could not clearly express what made her anxious. Her crying and sadness happened suddenly, without obvious reasons, and she even denied low mood sometimes and said she had come to the hospital for cardiac disease treatment (she did not have any cardiac disease). Her description of her depressed mood was uncertain when specifically questioned, and she rarely reported her depressed feeling spontaneously as other depressed patients would. She did not even have the desire to get rid of her “depression”. Her suicidal ideation was transient and impulsive, and she could not provide a comprehensive explanation for her suicide attempts, such as emptiness, worthlessness or guilt. She was impatient and restless when interacting with others or when a more in-depth conversation was performed. She seemed apathetic, gave little response to emotional support from others and did not care about relevant important issues, such as hospital discharge or future plans. Elevated mood and motor activity were not found during the admission period.
- Social withdrawal and inappropriate behaviors: The patient often walked or stayed alone for long periods of time before speaking to other patients suddenly, which seemed improper or even odd in normal social interactions. During most hospitalization periods, lethargy and withdrawal were obvious.

## Diagnostic assessment and therapeutic interventions

Basic laboratory tests reported abnormal results (Table 1), and the circulating cortisol level was far beyond the upper limit of normal, with a loss of circadian rhythm (Table 2); 24-h urinary free cortisol : >2897 nmol/24 h↑(69–345 nmol/24 h); serum ACTH (8 AM, 4 PM, 12 PM): 1.2 pg/ml, 1.3 pg/ml, < 1 pg/ml (normal range: 1–46 pg/ml); low-dose dexamethasone suppression test (1 mg) (cortisol value): 1010.1 nmol/l (not suppressed; normal range: <50 nmol/L); high dose dexamethasone inhibition test (cortisol value): 879.0 nmol/l (not suppressed); OGTT and glycosylated hemoglobin; both normal. Other results used to rule out hyperaldosteronism and pheochromocytoma, such as the aldosterone/renin rate (ARR) and the vanillylmandelic acid, dopamine, norepinephrine and epinephrine levels, were reported to be within normal limits; ECG suggested sinus tachycardia; dual-energy X-ray bone density screening values were lower than the normal range; B-mode ultrasound showed a right adrenal tumor and fatty liver. The abdominal CT scan showed a tumor in her right adrenal gland. Brain MRI showed no abnormalities. Psychometric tests including HAMD (Hamilton depression scale), MADRS (Montgomery-Asberg Depression Rating Scale), WAIS (Wechsler Intelligence Scale) and MMSE (Mini-mental State Examination) were hard to perform due to her poor attention and non-cooperation presentation.

**Table 1:** Abnormal lab results for the patient.

**Lab test**	**Result**	**Reference values**
Total cholesterol	6.36 mmol/L	1.00–5.22 mmol/L
LDL cholesterol	4.66 mmol/L	2.60–3.30 mmol/L
ALT	157.8 mg/L	0.00–49.0 mg/L
AST	42.4 mg/L	0.00–34.0 mg/L
Serum potassium	2.95 mmol/L	3.5–5.5 mmol/L
Testosterone	2.19 mmol/L	0.52–1.47 mmol/L
Androstenedione	18.30 nmol/L	4–6.6 nmol/L

**Table 2:** Circulating cortisol level.

**Time**	**Serum cortisol (ug/L)**	**Reference values (ug/L)**
8 AM	427.52	52.7–224.50
4 PM	409.7	34.4–167.6
12 PM	318.34	19.8–46

The patient had little response to adequate antidepressants in our hospital, including fluoxetine 20–60 mg/d and aripiprazole 5–30 mg/d combined with 3 sessions of MECT (modified electroconvulsive therapy), which was stopped because of her poor cognitive function and poor response.

Her last diagnosis was right adrenal adenoma and non-ACTH-dependent Cushing's syndrome. The adrenal adenoma was excised through laparoscopic resection in a general hospital. Hydrocortisone, amlodipine besylate, potassium chloride, metoprolol and escitalopram were used for treatment. Escitalopram 10 mg/d has been used until 2 weeks after her discharge. At the follow-up visit about 1 month after the surgery, her depressive mood had significantly improved, with no self-injury behaviors or psychiatric symptoms found. The patient was calm but still reacted slowly, and cognitive impairment was still found at the last visit.

## Discussion

Previous studies have reported a close association between CS and depression (15). However, suicidal depression with atypical features and acute psychosis have rarely been reported, and the characteristics of depression secondary to CS and the differences from major depression have not been described in detail.

This case did not show a full-blown presentation of major depression according to the DSM-5. She presented with a series of features that were not typical as major depression, however, it should be emphasized that the atypical features were not identical to those noted in DSM5, especially regarding increased appetite and hypersomnia. The features suggesting difference from major depression were listed as follows: (a) depressed mood is not constant, it does not exist in most of the day; it is episodic without regular cyclicity, can happen or exacerbate suddenly; (b) the ability to describe anhedonia is poor, she can't report her feeling voluntarily like other patients with major depression, which might be partially related with the decreased language function; (c) depressive thoughts such as self-accusation and feelings of guilt, the classical symptoms of major depression, were rarely found; (d) more exaggerated cognitive impairment and decrease language function; € partial or little useful effect of SSRIs (selective serotonin reuptake inhibitors). The above characteristics were similar to those reported in Starkman's research (13, 16, 17), in which increasing irritability was also regarded as one of the important features for depression in CS.

The literature about depression combined with psychosis episode in CS is rare. This patient showed acute episodes of persecutory delusion with disturbed behaviors; her psychotic symptoms occurred suddenly and were fragmentary, with poor sensitivity to antipsychotics; the content was not constant (she never referred to and even denied the unsafe feeling at home before admission), it changed with the environment and was not consistent with the mood state. However, we cannot reach an effective conclusion because the evidence was small; thus, these findings should be evaluated in combination with other clinical presentations.

## Conclusion

Most reviews have concluded that mood disturbances in CS indicate “major depression”, but the detailed description of clinical features are lack, making clinicians uncertain about the presentation and confused about the diagnosis, especially when the somatic signs are indiscriminate. The clinical presentation in this case highlighted the fact that there is a wide range of phenotypes of depression in CS, for some CS patients, the depressive features are not highly consistent with the criteria of major depression regardless of the melancholic or atypical features in the DSM-5. Thus, a thorough and periodic evaluation is necessary to detect the underlying organic and psychosocial causes if the clinical symptoms are not typical (10).

## Data availability statement

The original contributions presented in the study are included in the article/supplementary material, further inquiries can be directed to the corresponding author.

## Ethics statement

Written informed consent was obtained from the individual(s), and minor(s)' legal guardian/next of kin, for the publication of any potentially identifiable images or data included in this article.

## Author contributions

XY, SC, XJ, and XH were responsible for clinical care. XY did literature search and drafted the manuscript. XH revised the manuscript. All authors contributed to the article and have approved the final manuscript.
